# Chemical profile and antiparasitic effects of *Lippia origanoides* essential oil against *Rhipicephalus microplus*, *Haemonchus contortus*, and *Caenorhabditis elegans*

**DOI:** 10.1590/S1984-29612025072

**Published:** 2025-12-12

**Authors:** Joelson Gomes de Oliveira, Caio Pavão Tavares, Tássia Lopes do Vale, Dauana Mesquita-Sousa, Gabriel Sousa Brito, Márcia Aldeany Almeida de Sousa, José Fábio França Orlanda, Odair dos Santos Monteiro, Wesley Douglas Ribeiro, Lívio Martins Costa-Junior, Francisco Eduardo Aragão Catunda, José Roberto Pereira de Sousa

**Affiliations:** 1 Universidade Estadual do Maranhão – UEMA, Centro de Ciências Agrárias, São Luís, MA, Brasil; 2 Universidade Federal do Maranhão – UFMA, Centro de Ciências Biológicas e da Saúde, São Luís, MA, Brasil; 3 Universidade Estadual da Região Tocantina do Maranhão – UEMASUL, Centro de Ciências Exatas, Naturais e Tecnológicas, Imperatriz, MA, Brasil; 4 Universidade Federal do Maranhão – UFMA, Centro de Ciências Exatas e Tecnológicas, Departamento de Química, São Luís, MA, Brasil

**Keywords:** Cattle tick, camphor, egg hatch assay, helminth, lethal concentration, Carrapato bovino, cânfora, ensaio de eclosão de ovos, helminto, concentração letal

## Abstract

Growing antiparasitic resistance challenges parasite control in livestock, increasing interest in natural product-based alternatives for animal health and food quality. This study evaluated the *in vitro* activity of *Lippia origanoides* essential oil against *Rhipicephalus microplus*, *Haemonchus contortus*, and *Caenorhabditis elegans*. Fresh leaves of *L. origanoides* from Maranhão, Brazil, were used to obtain essential oil by hydrodistillation, and its chemical composition was determined by gas chromatography–mass spectrometry (GC–MS). Bioassays evaluated activity against *R. microplus* larvae (immersion and repellency), *H. contortus* eggs (hatch inhibition), and *C. elegans* adults (mortality), all using serial dilutions of essential oil. Experiments were performed in nine replicates, and results were analyzed by nonlinear regression. Chemical analysis of the oil revealed the main constituents: camphor (32.13%), β-bisabolene (10.02%) and camphene (6.66%). The larval immersion test of the oil showed an LC_50_ (letal concentration) of 7.48 mg/mL against ticks. A repellency assay revealed an RC_50_ (repellent concentration) of 0.058 mg/cm^2^ at 10 minutes. The egg hatch assay for *H. contortus* resulted in an LC_50_ of 0.67 mg/mL, whereas the adult mortality assay for *C. elegans* showed an LC_50_ of 2.23 mg/mL. These results suggest the acaricidal, repellent, and nematicidal efficacy of essential oil of *L. origanoides*.

## Resumo

A crescente resistência antiparasitária dificulta o controle de parasitos em animais de produção, aumentando o interesse por alternativas baseadas em produtos naturais para a saúde animal e a qualidade dos alimentos. Este estudo avaliou a atividade *in vitro* do óleo essencial de *Lippia origanoides* contra *Rhipicephalus microplus*, *Haemonchus contortus* e *Caenorhabditis elegans*. Folhas frescas de *L. origanoides* foram utilizadas para obtenção do óleo essencial por hidrodestilação e sua composição química foi determinada por cromatografia gasosa–espectrometria de massa. Bioensaios avaliaram a atividade contra larvas de *R. microplus* (imersão e repelência), ovos de *H. contortus* (inibição da eclosão) e adultos de *C. elegans* (mortalidade). Os experimentos foram realizados em nove repetições. A análise química revelou como principais constituintes: cânfora (32,13%), β-bisaboleno (10,02%) e cânfeno (6,66%). O teste de imersão larval mostrou uma CL_50_ (concentração letal) de 7,48 mg/mL contra carrapatos. Um ensaio de repelência revelou uma CR_50_ (concentração repelente) de 0,058 mg/cm^2^ aos 10 minutos. O ensaio de eclosão de ovos para *H. contortus* resultou em uma CL_50_ de 0,67 mg/mL, enquanto o ensaio de mortalidade de *C. elegans* mostrou uma CL_50_ de 2,23 mg/mL. Esses resultados sugerem a eficácia acaricida, repelente e nematicida do óleo essencial de *L. origanoides*.

## Introduction

Brazil holds a prominent position in global livestock production, being the second-largest producer of cattle and beef. According to USDA data, the Brazilian herd has reached 264 million head, with a production of 9.75 million tons of beef (Formigoni, 2022). However, livestock farming faces significant challenges related to parasite control, especially the tick *R. microplus*, an ectoparasite that affects cattle, resulting in annual losses exceeding US$ 3 billion (Grisi et al., 2014; Garcia et al., 2019).

The farming of goats and sheep is also widely practiced in the country, with the Northeast region accounting for 94.5% of goats and 68.5% of sheep (Magalhães et al., 2020). In this sector, the main damages are associated with gastrointestinal parasitosis caused by nematodes from the Trichostrongyloidea family, especially *Haemonchus contortus*, a blood-feeding abomasal parasite responsible for severe anemia, weight loss, and reduced productivity of meat, milk, leather, and wool (Amarante, 2014; Besier et al., 2016; Almeida et al., 2018). Global losses caused by *H. contortus* are estimated at US$ 300 million per year (Emery et al., 2016; Ehsan et al., 2020). Since the direct assessment of this parasite requires host slaughter, the free-living nematode *C. elegans* has been used as an *in vitro* experimental model due to its morphological and physiological similarities to parasitic nematodes, short life cycle, high reproductive rate, and well-characterized genome (Katiki et al., 2017).

The control of these parasitosis is traditionally carried out using synthetic acaricides and anthelmintics. However, the extensive and sometimes indiscriminate use of these products has led to the emergence of resistant parasite populations (Heylen et al., 2024; Salvador et al., 2025; Ferreira et al., 2025), in addition to raising concerns about residues in animal products and environmental impacts. In this context, there is an urgent need to seek alternative control strategies that are effective, sustainable, and safe for animals, consumers, and the environment.

Among these strategies, the use of plant-derived natural products stands out, especially essential oils, which exhibit a wide range of biological activities, including repellent, acaricidal, and anthelmintic properties. *Lippia origanoides* (Verbenaceae) is an aromatic plant species widely distributed in the Americas, and its essential oil has been extensively investigated, revealing a broad spectrum of biological activities, ranging from antioxidant (Teles et al., 2014; Damasceno et al., 2018) to antiparasitic effects (Soares et al., 2017; Castro et al., 2018). Compounds frequently found in the *Lippia* genus include thymol, carvacrol, 1,8-cineole, limonene, *p*-cymene, linalool, α-pinene, β-caryophyllene, camphor, and camphene (Oliveira et al., 2007; Stashenko et al., 2008; Sousa et al., 2020).

In this context, this study aimed to evaluate the acaricidal, repellent, and nematicidal activities of the essential oil extracted from fresh leaves of *L. origanoides* Kunth “camphor chemotype” against the tick *R. microplus* and the nematodes *H. contortus* and *C. elegans* under laboratory conditions, as well as to chemically characterize the oil composition.

## Material and Methods

### Plant material

Leaves, branches, and inflorescences of *L. origanoides* Kunth were collected from mature plants naturally occurring in the municipality of Montes Altos (5°50’0.63”S 47°16’9.60”W), state of Maranhão, Brazil. Sampling was carried out between 8:00 and 10:00 a.m. in the period of January to March 2021. Voucher specimens were deposited at the herbarium of the State University of the Tocantina Region of Maranhão (UEMASUL), under registration numbers 209, 210, and 211.

### Extraction of essential oil

Fresh leaves were washed first in running water, then in distilled water, after which they were cut into smaller pieces for oil extraction. The essential oil (EO) was extracted by hydrodistillation at 100 °C for three hours, using a modified Clevenger-type apparatus. For each extraction, 100 g of fresh plant material was mixed with 1000 mL of water in a 1:10 ratio. The extracted oil was stored in amber glass vials wrapped in aluminum foil under refrigeration (2 to 8ºC) to avoid losses due to volatilization (Sousa et al., 2020).

### Chemical characterization of the essential oil

A chemical analysis was conducted at the Natural Products Chemistry Laboratory of the State University of Pará (UEPA), Brazil. A qualitative analysis was performed using gas chromatography coupled to mass spectrometry (GC-MS) on a Shimadzu QP 2010 Ultra system, with a 1 µL injection of a 3:500 oil-in-hexane solution (Auto Injector AOC-20i). A Rtx-5MS silica capillary column (Restek, USA) was used, with a 30 m length × 0.25 mm internal diameter, coated with 5%-diphenyl/95%-dimethylpolysiloxane (0.25 µm film thickness).

The oven temperature was programmed from 60 °C to 240 °C (10 min hold) at an incremental rate of 3 °C/min. The injector (split ratio 1:20), transfer line, and ion source were set at 250 °C, 250 °C, and 200 °C, respectively. Helium was used as the carrier gas at a flow rate of 1 mL/min. Mass spectra were obtained by electron impact at 70 eV, with automatic scanning in the range of 35 to 400 Daltons at a rate of 0.30 scans/s. Compound identification was based on linear retention indices (calculated using a series of n-alkanes, C8–C40) and mass spectra were compared with spectra in the Adams (2006), NIST 2011, and FFNSC 2 libraries.

### Tick population

Engorged *R. microplus* females were collected from calves artificially infested at the Central Vivarium of the Federal University of Maranhão – UEMA at São Luís, MA, Brazil. The engorged females were washed in distilled water, dried with filter paper, placed in Petri dishes, and kept in a B.O.D. incubator at 27 ± 1 °C with relative humidity (RH) ≥ 80% until oviposition was complete. Eggs were collected, transferred to 15 mL Falcon tubes sealed with cotton, and incubated until hatching. Larval immersion and repellency tests were carried out using 14 to 21-day-old larvae.

### Larval Immersion Test (LIT)

The efficacy of the EO against *R. microplus* larvae was evaluated using the Larval Immersion Test (LIT) described by Klafke et al. (2006). EO was diluted in a solution of 1.0% ethanol and 0.02% Triton X-100. The control group was treated with the same ethanol-Triton solution. EO efficacy was tested at concentrations of 10.0, 7.0, 4.90, 3.43, 2.40, 1.68, 1.17, 0.82, 0.57, and 0.40 mg/mL. Approximately 500 larvae were immersed in each solution for 10 minutes at 25 °C, after which they were transferred to filter paper for drying. Around 100 larvae were then placed on clean, dry filter paper (8.5 × 7.5 cm), which was folded and stapled to form a packet. The packets were incubated at 27 ± 1 °C and RH ≥ 80% for 24 hours. After incubation, live and dead larvae were counted using a vacuum pump. Larvae that were immobile or exhibited morphological alterations (e.g., shrinkage) were considered dead. The experiment was performed with nine replicates per treatment. The median lethal concentration (LC_50_) was subsequently calculated.

The essential oil concentrations were defined based on previous studies conducted by our research group with natural products. This strategy aims not only at the bioprospecting of bioactive compounds but also at meeting preliminary requirements of the veterinary products industry, which tends to disregard investigations reporting excessively high effective concentrations due to economic constraints.

### Repellency test

The repellency test (vertical filter paper bioassay) was carried out applying the method described by Carroll et al. (2004), using 7 × 4 cm QM quantitative filter paper. The central area (5 × 4 cm) was treated with 165 µL of EO at various concentrations (1.25; 0.625; 0.312; 0.156; 0.078; 0.039; 0.019; 0.001 mg/mL). DEET, a well-established repellent, was employed as the positive control in the repellency assays, tested at the same concentrations previously described (1.25, 0.625, 0.312, 0.156, 0.078, 0.039, 0.019, and 0.001 mg/mL). The upper and lower 1 × 4 cm areas remained untreated (neutral zones). The selection of oil concentrations for the assays followed the same rationale presented in the “Larval Immersion Test (LIT)” section.

The filter paper was dried at room temperature for 10 minutes and then suspended vertically by one of the untreated ends on wooden sticks. Approximately 100 larvae were placed on the lower untreated portion of each filter paper, and the distribution of larvae throughout the paper was recorded at 10, 20 and 30 minutes post-drying. Distilled water was used as the negative control. All the tests were performed in nine replicates. Larvae that moved away from the treated central zone and remained in the untreated areas were considered repelled. Distilled water was used as the negative control. Based on the percentage of larvae avoiding the treated zone, the repellency concentration required to repel 50% of the larvae (RC_50_) was calculated.

### Maintenance of *Haemonchus contortus* strain and Egg Hatch Assay (EHA)

Crossbreading sheep (18 months old) were artificially infected with a monospecific population of *Haemonchus contortus* through the oral administration of 5,000 third-stage larvae (L3). This animal was kept at the Federal University of Maranhão (UFMA).

*Haemonchus contortus* eggs were collected 21 days after infection with L3 larvae and obtained by washing feces through successive sieves of different mesh sizes (1 mm, 105 μm, 55 μm, and 25 μm). The collection followed the method described by Hubert & Kerboeuf (1992). A solution containing 1000 eggs/mL was prepared, and 100 μL of this solution (approximately 100 eggs) was placed into the wells of a flat-bottom 96-well plate (Cralplast, Cotia, SP, Brazil). Three replicates of 100 μL of the *L. origanoides* essential oil dilutions (12 concentrations ranging from 10 to 0.005 mg/mL) were added to each well. The final concentration of DMSO in each well was 1%, and Tween-80 was 0.0015%. The selection of oil concentrations for the assays followed the same rationale presented in the “Larval Immersion Test (LIT)” section.

A negative control containing the respective solvents at these concentrations was included. The plate was incubated at 27 °C and relative humidity (RH) > 80%. After 48 h, eggs and larvae were quantified under an inverted microscope (Primovert, Carl Zeiss, Oberkochen, Germany) (Coles et al., 1992). The assays, including negative controls, were repeated in three independent experiments, each with three replicates.

### Maintenance of *Caenorhabditis elegans* strains and mortality test

The *C. elegans* Bristol N2 strain was donated by Vale do Acaraú State University (UVA), Brazil, and maintained in the Parasite Control Laboratory (LCP) at UFMA, Brazil. The nematodes were cultured on Nematode Growth Medium (NGM), which was seeded with *Escherichia coli* strain NA22 under standard conditions (Brenner, 1974; James & Davey, 2009).

Four-day-old Petri dishes containing *C. elegans* cultures were used to isolate juvenile and adult worms. The nematodes were recovered using M9 buffer and isolated with granulometric sieves measuring 38 µm and 53 µm, as described by Ferreira et al. (2015). The recovered nematodes were counted and adjusted to a concentration of 100 nematodes per 100 µL of M9 buffer. The EO was diluted with 3% Tween 80 to obtain the stock solution. The stock solution was then diluted in 96-well plates using 50% serial dilutions in 12 concentrations with a range of 10 to 0.005 mg/mL. 100 µL of M9 buffer containing approximately 100 nematodes was placed in each well. All the concentrations were performed in nine replicates. Negative controls consisted of M9 with 3% Tween 80. The plates were incubated at 24 °C for 24 hours. After incubation, live and dead nematodes were counted under an inverted microscope (Carl Zeiss, Baden-Württemberg, Germany). Nematodes that remained immobile for five seconds were considered dead (Katiki et al., 2011).

The selection of oil concentrations for the assays followed the same rationale presented in the “Larval Immersion Test (LIT)” section.

### Statistical analysis and ethics statement

To determine the LC_50_ based on the LIT and the RC_50_ on the Repellency Test, EO concentrations were log-transformed and normalized. Nonlinear regression analysis was conducted using GraphPad Prism 8.0 (GraphPad Inc., San Diego, CA, USA).

The lethal concentration (LC_50_) and the effective concentration (EC_50_) for the *C. elegans* mortality and egg hatch inhibition tests, respectively, were calculated according to the method proposed by Roditakis et al. (2005).

The coefficient of determination (R^2^) was used to evaluate the quality of the regression model fit to the experimental data. R^2^ values greater than 0.70 were considered indicative of a good fit, whereas values close to 1.0 reflected a strong explanatory capacity of the model to describe the relationship between the substance concentration (independent variable) and the observed biological response (dependent variable).

This study was approved by the Ethics Committee on Animal Experimentation at UFMA under protocol number 23115.002637/2023-43.

## Results

### Chemical characterization of the essential oil

GC-MS analysis allowed the identification of 90.52% of the chemical constituents of the EO of *Lippia origanoides* (Table 1). Oxygenated monoterpenes were the predominant class (40.77%), with camphor (32.13%) as the major compound, followed by borneol (4.11%) and 1,8-cineole (1.25%). The second most abundant group was hydrocarbon sesquiterpenes (22.77%), mainly represented by β-bisabolene (10.02%) and α-E-bergamotene (2.43%). Oxygenated sesquiterpenes accounted for 15.93% of the composition, with caryophyllene oxide (3.36%), pogostol (3.05%), and elemol (1.92%) as the most relevant constituents. Hydrocarbon monoterpenes represented 10.28% of the total, with camphene (6.66%) as the most abundant. Minor constituents included phenylpropanoids (0.18%) and other compounds such as acetophenone and farnesyl acetate (0.59%).

**Table 1:** Chemical composition of essential oil from leaves of *Lippia origanoides* Kunth “Camphor Chemotype”.

**Class**	**Compound**	**Retention Time (min)**	**% Area**
**Monoterpenes (Hydrocarbons)**	Tricyclene	5.548	0.04
	α-Pinene	5.834	0.95
	Camphene^3^	6.244	6.66
	Sabinene	6.912	0.15
	β-Pinene	7.030	0.82
	Myrcene	7.385	0.29
	*p*-Cymene	8.505	0.13
	Limonene	8.660	1.17
	Camphene hydrate	13.271	0.07
**Subtotal**			**10.28**
**Monoterpenes (Oxygenated)**	1,8-Cineole	8.748	1.25
	Linalool	11.247	0.09
	Camphor^1^	13.163	32.13
	Borneol	13.978	4.11
	α-Terpineol	14.999	0.36
	Verbenone	15.767	0.31
	E-Carveol	16.187	0.45
	Carvone	17.239	1.15
	cis-Sabinene hydrate	10.022	0.07
	cis-Limonene oxide	12.513	0.08
	p-Cymen-8-ol	14.749	0.57
	Myrtenol	15.252	0.20
**Subtotal**			**40.77**
**Sesquiterpenes (Hydrocarbons)**	α-Copaene	22.930	0.81
	β-Elemene	23.603	0.78
	E-Caryophyllene	24.739	1.83
	α-E-Bergamotene	25.407	2.43
	α-Humulene	26.124	0.23
	Z-β-Farnesene	26.247	0.36
	9-epi-E-Caryophyllene	26.417	0.54
	β-Selinene	27.570	0.96
	β-Bisabolene^2^	28.385	10.02
	δ-Cadinene	28.937	0.45
	(E)-α-Bisabolene	29.688	1.39
	β-Bourbonene	23.304	0.06
	Sesquithujene	24.174	0.06
	α-Guaiene	25.523	0.16
	ar-Curcumene	27.293	0.73
	γ-Cadinene	28.616	0.22
	α-Copaen-11-ol	29.600	0.06
	α-Cedrene	24.430	0.28
	γ-Muurolene	27.068	0.11
	α-Bulnesene	27.988 / 28.245	0.52
	α-Alaskene	28.528	0.29
	cis-Calamenene	29.311	0.22
**Subtotal**			**22.77**
**Sesquiterpenes (Oxygenated)**	Guaiol	31.800	0.06
	γ-Eudesmol	33.086	0.24
	β-Eudesmol	33.757	0.51
	epi-β-Bisabolol	34.489	1.39
	α-Bisabolol	34.982	0.33
	epi-Cubebol	27.782	0.19
	Elemol	29.929	1.92
	Spathulenol	31.034	0.47
	Caryophyllene oxide	31.246	3.36
	Pogostol	33.918	3.05
	Shyobunol	35.256	0.47
	Ledol	32.008	0.63
	β-Atlantol	32.213	1.74
	Muurola-4,10(14)-dien-1-β-ol	32.960	0.41
	epi-α-Muurolol	33.460	0.22
	α-Muurolol	33.630	0.29
	cis-Calamenen-10-ol	34.082	0.35
	Eudesm-7(11)-en-4-ol	35.798	0.15
	Cryptomerione	36.555	0.08
	Oplopanone	36.913	0.07
**Subtotal**			**15.93**
**Phenylpropanoids**	Methyl chavicol	15.321	0.12
	E-Anethole	19.040	0.06
**Subtotal**			**0.18**
**Others**	Acetophenone	9.967	0.07
	(2E,6E)-Farnesyl acetate	40.512	0.32
	2E,6Z-Farnesal	36.065	0.10
	2E,6E-Farnesol	37.069	0.10
**Subtotal**			**0.59**
**Total Identified**			**90.52**

Note: %: percentage of each chemical constituent. Numbers 1, 2, and 3: ranking of the main compounds.

**Figure 1:**
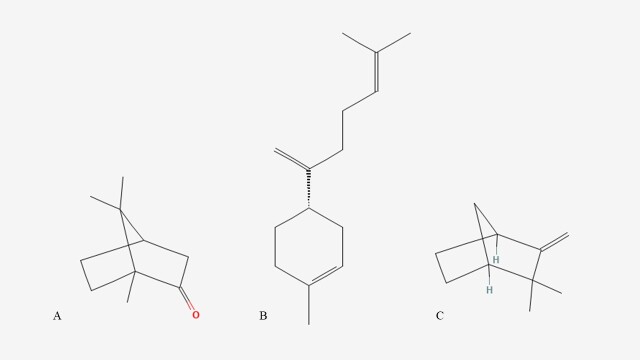
Chemical structures of the three main components of *Lippia origanoides* Kunth “Camphor Chemotype” essential oil. (A) camphor; (B) β-bisabolene; (C) camphene.

Overall, the chemical profile of the EO reveals a composition rich in oxygenated monoterpenes and sesquiterpenes, with camphor as the predominant marker of the analyzed chemotype. The chemical structures of the three main components (camphor, β-bisabolene, and camphene) are shown in Figure 1.

### Efficacy against *Rhipicephalus microplus*

The EO of *L. origanoides* demonstrated significant activity against *R. microplus* larvae. In the larval immersion test (LIT), the EO exhibited larvicidal properties, with an LC_50_ value of 7.48 mg/mL (95% CI: 7.12–7.85; R^2^ = 0.96). In the larval repellency assay, the negative control consistently showed repellency rates below 10%. In contrast, the EO exhibited repellent effects at all tested concentrations, with RC_50_ values of 0.058, 0.101, and 0.310 mg/cm^2^ at 10, 20 and 30 minutes post-application, respectively. However, despite demonstrating dose-dependent repellency, the EO was less potent than DEET, the positive control (Table 2). The mean percentages of mortality and repellency of *R. microplus* larvae are presented in Table 3.

**Table 2:** Efficacy of the essential oil of *Lippia origanoides* Kunth “Camphor Chemotype” against *Rhipicephalus microplus* larvae, *Haemonchus contortus* eggs, and adult *Caenorhabditis elegans*.

**Biological assay/Parasite**	**Treatment**	**Parameters**			
**Larval Repellency Test**		**Time (min)**	**RC_50_ (mg/cm^2^)**	**95% CI**	**R^2^**
*R. microplus*		10	0.025	0.021-0.030	0.98
	DEET	20	0.026	0.019-0.030	0.96
		30	0.031	0.026-0.037	0.96
		10	0.058	0.051-0.067	0.92
	*L. origanoides*	20	0.101	0.089-0.116	0.95
		30	0.310	0.282-0.338	0.97
**LIT**		**LC_50_ (mg/mL)**			
*R. microplus*	*L. origanoides*	7.482		7.121-7.852	0.96
**Egg hatch inhibition**		**EC_50_ (mg/mL)**			
*H. contortus*	*L. origanoides*	0.671		0.601-0.742	0.97
**Mortality**		**LC_50_ (mg/mL)**			
*C. elegans*	*L. origanoides*	2.232		1.792-2.788	0.91

Note: RC_50_, concentration at which 50% of *R. microplus* larvae were repelled (mg/cm^2^); LC_50_, concentration at which 50% of *R. microplus* or *C. elegans* larvae were killed (mg/mL); EC_50_, concentration at which 50% of *H. contortus* eggs failed to hatch (mg/mL); 95% CI, confidence interval; R^2^, coefficient of determination.

**Table 3:** Percentage (mean ± standard error) of mortality and repellency of *Rhipicephalus microplus* larvae treated with different concentrations of *Lippia origanoides* Kunth essential oil 'Camphor Chemotype'.

**Larval Immersion Test**		**Repellency Test (%)**			
**mg/mL**	**Mortality %**	**mg/cm^2^**	**10 min**	**20 min**	**30min**
**10.0**	100.00 ± 0.00	**1.650**	97.01 ± 0.00	89.90 ± 0.00	80.22 ± 0.00
**7.00**	25.37 ± 7.09	**0.625**	83.42 ± 0.00	74.45 ± 0.00	62.19 ± 0.00
**4.90**	7.71 ± 1.40	**0.312**	79.78 ± 0.00	55.42 ± 0.00	51.87 ± 0.00
**3.430**	0.00 ± 0.00	**0.156**	77.02 ± 0.00	57.44 ± 0.00	42.01 ± 0.00
**2.401**	0.00 ± 0.00	**0.078**	63.21 ± 0.00	46.53 ± 0.00	38.43 ± 0.00
**1.680**	0.00 ± 0.00	**0.039**	44.35 ± 0.00	31.01 ± 0.00	29.16 ± 0.00
**1.176**	0.00 ± 0.00	**0.019**	21.03 ± 0.00	17.09 ± 0.00	10.02 ± 0.00
**0.824**	0.00 ± 0.00	**0.001**	13.74 ± 0.00	9.54 ± 0.00	3.06 ± 0.00
**0.576**	0.00 ± 0.00	**-**	-	-	-
**0.403**	0.00 ± 0.00	**-**	-	-	-

### Anthelmintic activity against *Haemonchus contortus* and *Caenorhabditis elegans*

The EO of *L. origanoides* also exhibited ovicidal and anthelmintic effects against gastrointestinal nematodes. In the egg hatch assay (EHA) using *H. contortus*, the EO inhibited embryonic development in a dose-dependent manner, with an EC_50_ value of 0.67 mg/mL (95% CI: 0.60–0.74; R^2^ = 0.97). Additionally, in the adult mortality assay with *C. elegans* (strain Bristol N2), the EO showed anthelmintic activity, with an LC_50_ value of 2.23 mg/mL (95% CI: 1.79–2.78; R^2^ = 0.91) (Table 2). The mean percentages of egg hatching of *H. contortus* and mortality of *C. elegans* are presented in Table 4.

**Table 4:** Percentage (mean ± standard error) of egg hatching of *Haemonchus contortus* and mortality of adult *Caenorhabditis elegans* treated with different concentrations of *Lippia origanoides* Kunth essential oil 'Camphor Chemotype'.

**Egg Hatch Assay (*H. contortus*)**		**Mortality test (*C. elegans*)**	
**mg/mL**	**Hatchability %**	**mg/mL**	**Mortality %**
**10.00**	100.00 ± 0.00	**10.00**	100.00 ± 0.00
**5.000**	100.00 ± 0.00	**5.000**	63.00 ± 13.08
**2.500**	100.00 ± 0.00	**2.500**	58.33 ± 6.43
**1.250**	70.00 ± 4.58	**1.250**	34.33 ± 9.24
**0.625**	57.33 ± 10.07	**0.625**	17.33 ± 5.13
**0.313**	2.00 ± 3.46	**0.313**	11.67 ± 7.37
**0.156**	0.00 ± 0.00	**0.156**	0.00 ± 0.00
**0.078**	0.00 ± 0.00	**0.078**	0.00 ± 0.00
**0.039**	0.00 ± 0.00	**0.039**	0.00 ± 0.00
**0.020**	0.00 ± 0.00	**0.020**	0.00 ± 0.00
**0.010**	0.00 ± 0.00	**0.010**	0.00 ± 0.00
**0.005**	0.00 ± 0.00	**0.005**	0.00 ± 0.00

## Discussion

This study presents novel findings on the acaricidal and nematicidal activity of the EO from *L. origanoides*, camphor chemotype, against *R. microplus*, *H. contortus*, and *C. elegans*. The originality of the research lies in the simultaneous assessment of the acaricidal, repellent, and nematicidal properties of this specific chemotype, whose chemical composition was thoroughly characterized.

The chemical analysis of the EO revealed a greater number of compounds than those reported by Tozin et al. (2015) and Ribeiro et al. (2021) (28 and 49 substances, respectively), but fewer than the 139 compounds described by Stashenko et al. (2004). The compositional similarity with the *L. origanoides* sample analyzed by Sousa et al. (2020), also collected in Montes Altos, MA, suggests that both belong to the same chemotype, characterized by camphor (34.04%), camphene (10.99%), and β-bisabolene (10.8%) as major constituents. This profile was also observed in samples from Minas Gerais by Souza et al. (2019). Chemotypic variations are attributed to genetic, environmental, methodological factors, and the phenological stage of the plant (Stashenko et al., 2004; Rojas et al., 2006; Heinzmann et al., 2017), and at least five chemotypes have been described for *L. origanoides* (Sousa et al., 2020).

The acaricidal activity observed in this study corroborates previous findings highlighting the potential of monoterpenic compounds such as camphor, already recognized for its efficacy against *R. microplus* (Yang et al., 2021). In that study, both the EO and isolated camphor achieved 100% larval mortality at 1.6% (v/v) and approximately 70% mortality in engorged females. Similarly, Kapoor & Preet (2023) demonstrated the acaricidal efficacy of a nanoemulsion of *Cinnamomum camphora* (20.89% camphor), with an LC_50_ of 3.36 mg/mL. Although more potent than the EO tested herein, these findings suggest that nanotechnological formulations may enhance the potency of *L. origanoides* EO. Camphor has also exhibited high toxicity against house dust mites such as *Dermatophagoides* spp., outperforming benzyl benzoate (Yang & Lee, 2013), especially when in synergy with α-pinene (Volpato et al., 2015).

Regarding camphene and β-bisabolene, available data remain scarce. Camphene showed weak acaricidal activity against *Tetranychus urticae* (Badawy et al., 2010), despite strong acetylcholinesterase inhibition. β-bisabolene, in turn, demonstrated promising results in formulations with *Copaifera officinalis* oil, inhibiting 100% of *R. microplus* larval hatching (Volpato et al., 2015). The combination of these compounds may thus confer relevant synergism, as previously suggested by Peixoto et al. (2015).

With regard to repellent activity, the tested essential oil showed relevant effects against *R. microplus* larvae, a species that can be used as a biological model for preliminary investigations. These results suggest potential application against the brown dog tick (*Rhipicephalus sanguineus* sensu lato), for which repellent products are more suitable in practical terms. Although few studies have explored camphor’s repellent effects against ticks, its efficacy against mosquitoes is well established (Omolo et al., 2004; Rehman et al., 2014; Haris et al., 2023). Camphor has also shown significant activity against the tick *Ixodes ricinus* (Pålsson et al., 2008) and the beetle *Tribolium castaneum* (Obeng-Ofori et al., 1998). Regarding camphene, available data are still limited but point to moderate repellent effects (Omolo et al., 2004; Feng et al., 2019).

Concerning ovicidal activity, the EO effectively inhibited egg hatching of *H. contortus*, performing comparably to other essential oils reported in the literature, such as *Eucalyptus globulus*, *Citrus aurantifolia*, *Rosmarinus officinalis*, and *Artemisia lancea* (Macedo et al., 2009; Ferreira et al., 2018; Aouadi et al., 2021; Zhu et al., 2013). Although camphor was the major compound, its isolated activity was low, suggesting a synergistic mechanism of action. This hypothesis is supported by similar effects observed with other *Lippia* species, such as *L. domingensis*, *L. alba*, and *L. sidoides*, which contain different major compounds but exhibit potent ovicidal activity (Camurça-Vasconcelos et al., 2007; Barbosa et al., 2023; Espino Ureña et al., 2023).

The experimental model using *C. elegans* confirmed the nematicidal activity of the EO, with an LC_50_ of 2.23 mg/mL. This is the first report of *L. origanoides* EO evaluated in this model. Given the relevance of *C. elegans* in toxicological tests and in the screening of broad-spectrum anthelmintic candidates (Kumarasingha et al., 2014; Nigon & Félix, 2017), the present findings provide valuable insights for future studies involving veterinary helminths, such as *H. contortus*.

For future studies, *in vivo* trials with EO-based formulations of *L. origanoides* are recommended, along with investigations into the molecular mechanisms underlying its acaricidal and nematicidal effects. Cytotoxicity evaluations using cellular models are also warranted. Ultimately, the findings suggest that this EO and its constituents hold potential for the development of new phytotherapeutic products targeting veterinary and agricultural parasites, particularly in light of increasing resistance to conventional chemical agents.

## Conclusions

The EO of *L. origanoides* contained camphor, β-bisabolene, and camphene as major components, exuding a soft camphor odor, which possibly represents a new chemotype for the species, as suggested in the literature.

According to the results obtained in this study, EO of *L. origanoides* Kunth “Camphor Chemotype” has acaricidal and repellent effects against *R. microplus*, and anthelmintic activity against *H. contortus* and *C. elegans*, showing greater promise in inhibiting *H. contortus* egg hatching, where the best results were obtained.

Overall, *L. origanoides* EO showed satisfactory activity and can be considered a potential candidate for further studies aimed at the safety and development of bioactive formulations for the control of ecto- and endoparasites.

## Data Availability

All data are available within the manuscript itself.

## References

[B001] Adams RP (2006). Identification of essential oils components by gas chromatography/mass spectrometry..

[B002] Almeida FA, Piza MLST, Bassetto CC, Starling RZC, Albuquerque ACA, Protes VM (2018). Infection with gastrointestinal nematodes in lambs in different integrated crop-livestock systems (ICL). Small Rumin Res.

[B003] Amarante AFT (2014). Sustainable worm control practices in South America. Small Rumin Res.

[B004] Aouadi M, Sebai E, Saratsis A, Kantzoura V, Saratsi K, Msaada K (2021). Essential oil of *Rosmarinus officinalis* induces *in vitro* anthelmintic and anticoccidial effects against *Haemonchus contortus* and *Eimeria* spp. in small ruminants. Vet Med.

[B005] Badawy MEI, El-Arami SAA, Abdelgaleil SAM (2010). Acaricidal and quantitative structure activity relationship of monoterpenes against the two-spotted spider mite, *Tetranychus urticae.*. Exp Appl Acarol.

[B006] Barbosa MLF, Ribeiro WLC, Araújo JV, Pereira RCA, André WPP, Melo ACFL (2023). In vitro anthelmintic activity of Lippia alba essential oil chemotypes against Haemonchus contortus.. Exp Parasitol.

[B007] Besier RB, Kahn LP, Sargison ND, Van Wyk JA (2016). The pathophysiology, ecology and epidemiology of *Haemonchus contortus* infection in small ruminants. Adv Parasitol.

[B008] Brenner S (1974). The genetics of *Caenorhabditis elegans.*. Genetics.

[B009] Camurça-Vasconcelos AL, Bevilaqua CM, Morais SM, Maciel MV, Costa CT, Macedo IT (2007). Anthelmintic activity of *Croton zehntneri* and *Lippia sidoides* essential oils. Vet Parasitol.

[B010] Carroll JF, Solberg VB, Klun JA, Kramer M, Debboun M (2004). Comparative activity of deet and AI3-37220 repellents against the ticks *Ixodes scapularis* and *Amblyomma americanum* (Acari: Ixodidae) in laboratory bioassays. J Med Entomol.

[B011] Castro KNC, Canuto KM, Brito ES, Costa-Júnior LM, Andrade IM, Magalhães JA (2018). In vitro efficacy of essential oils with different concentrations of 1,8-cineole against Rhipicephalus (Boophilus) microplus.. Rev Bras Parasitol Vet.

[B012] Coles GC, Bauer C, Borgsteede FHM, Geerts S, Klei TR, Taylor MA (1992). World Association for the Advancement of Veterinary Parasitology (W.A.A.V.P.) methods for the detection of anthelmintic resistance in nematodes of veterinary importance. Vet Parasitol.

[B013] Damasceno ETS, Almeida RR, Carvalho SYB, Carvalho GSG, Mano V, Pereira AC (2018). *Lippia origanoides* Kunth. essential oil loaded in nanogel based on the chitosan and ρ -coumaric acid: encapsulation efficiency and antioxidant activity. Ind Crops Prod.

[B014] Ehsan M, Hu RS, Liang QL, Hou JL, Song X, Yan R (2020). Advances in the development of anti-*Haemonchus contortus* vaccines: challenges, opportunities, and perspectives. Vaccines.

[B015] Emery DL, Hunt PW, Le Jambre LF (2016). *Haemonchus contortus*: the then and now, and where to from here?. Int J Parasitol.

[B016] Espino Ureña MJ, Katchborian-Neto A, Trinidad AB, Ramírez Ramírez M, Vásquez Tineo M, Araújo-Filho JV (2023). Chemical composition, anthelmintic activity, and mechanism of action of *Lippia dominguensis* Mold. essential oil on *Haemonchus contortus.*. Chem Biodivers.

[B017] Feng YX, Wang Y, Chen ZY, Guo SS, You CX, Du SS (2019). Efficacy of bornyl acetate and camphene from *Valeriana officinalis* essential oil against two storage insects. Environ Sci Pollut Res Int.

[B018] Ferreira SR, Mendes TAO, Bueno LL, Araújo JV, Bartholomeu DC, Fujiwara RT (2015). A new methodology for evaluation of nematode viability. BioMed Res Int.

[B019] Ferreira LE, Benincasa BI, Fachin AL, Contini SHT, França SC, Chagas ACS (2018). Essential oils of *Citrus aurantifolia, Anthemis nobile* and *Lavandula officinalis*: in vitro anthelmintic activities against *Haemonchus contortus.*. Parasit Vectors.

[B020] Ferreira PT, Bidone NB, Groff F, Silva PS, Jesus MS, Pellegrini DDCP (2025). Prevalence of and potential risk factors for multiple resistance to acaricides in *Rhipicephalus (Boophilus) microplus* ticks: A survey in the state of Rio Grande Do Sul, Brazil. Med Vet Entomol.

[B021] Formigoni I (2022). Farmnews: dados da produção mundial de carne bovina e por país, entre 2018 e 2022.

[B022] Garcia MV, Rodrigues VS, Koller WW, Andreotti R, Andreotti R, Garcia MV, Koller WW (2019). Carrapatos na cadeia produtiva de bovinos..

[B023] Grisi L, Leite RC, Martins JRS, Barros ATM, Andreotti R, Cançado PHD (2014). Reassessment of the potential economic impact of cattle parasites in Brazil. Rev Bras Parasitol Vet.

[B024] Haris A, Azeem M, Abbas MG, Mumtaz M, Mozūratis R, Binyameen M (2023). Prolonged repellent activity of plant essential oils against dengue vector, *Aedes aegypti.*. Molecules.

[B025] Heinzmann BM, Spitzer V, Simões CMO, Simões CMO, Schenkel EP, Mello JCP, Mentz LA, Petrovick PR (2017). Farmacognosia: do produto natural ao medicamento..

[B026] Heylen DJA, Labuschagne M, Meiring C, van der Mescht L, Klafke G, Costa LM (2024). Phenotypic and genotypic characterization of acaricide resistance in *Rhipicephalus microplus* field isolates from South Africa and Brazil. Int J Parasitol Drugs Drug Resist.

[B027] Hubert J, Kerboeuf D (1992). A microlarval development assay for the detection of anthelmintic resistance in sheep nematodes. Vet Rec.

[B028] James CE, Davey MW (2009). Increased expression of ABC transport proteins is associated with ivermectin resistance in the model nematode *Caenorhabditis elegans.*. Int J Parasitol.

[B029] Kapoor A, Preet S (2023). Evaluation of acaricidal activity of *Cinnamomum camphora* (F. lauraceae) essential oil nanoemulsion against cattle tick *Rhipicephalus microplus.*. Adv Zool Bot.

[B030] Katiki LM, Chagas ACS, Bizzo HR, Ferreira JFS, Amarante AFT (2011). Anthelmintic activity of *Cymbopogon martinii, Cymbopogon schoenanthus* and *Mentha piperita* essential oils evaluated in four different *in vitro* tests. Vet Parasitol.

[B031] Katiki LM, Barbieri AME, Araujo RC, Veríssimo CJ, Louvandini H, Ferreira JFS (2017). Synergistic interaction of ten essential oils against *Haemonchus contortus in vitro.*. Vet Parasitol.

[B032] Klafke GM, Sabatini GA, de Albuquerque TA, Martins JR, Kemp DH, Miller RJ (2006). Larval immersion tests with ivermectin in populations of the cattle tick *Rhipicephalus* (*Boophilus*) *microplus* (Acari: Ixodidae) from State of Sao Paulo, Brazil. Vet Parasitol.

[B033] Kumarasingha R, Palombo EA, Bhave M, Yeo TC, Lim DSL, Tu CL (2014). Enhancing a search for traditional medicinal plants with anthelmintic action by using wild type and stress reporter *Caenorhabditis elegans* strains as screening tools. Int J Parasitol.

[B034] Macedo ITF, Bevilaqua CML, Oliveira LMB, Camurça-Vasconcelos ALF, Vieira LS, Oliveira FR (2009). Atividade ovicida e larvicida in vitro do óleo essencial de *Eucalyptus globulus* sobre *Haemonchus contortus.*. Rev Bras Parasitol Vet.

[B035] Magalhães KA, Holanda ZF, Martins EC, Lucena CC. (2020). Caprinos e ovinos no Brasil: análise da produção da pecuária municipal 2019..

[B036] Nigon VM, Félix MA (2017). History of research on *C. elegans* and other free-living nematodes as model organisms. WormBook.

[B037] Obeng-Ofori D, Reichmuth CH, Bekele AJ, Hassanali A (1998). Toxicity and protectant potential of camphor, a major component of essential oil of *Ocimum kilimandscharicum*, against four stored product beetles. Int J Pest Manage.

[B038] Oliveira DR, Leitão GG, Bizzo HR, Lopes D, Alviano DS, Alviano CS (2007). Chemical and antimicrobial analyses of essential oil of *Lippia origanoides* H.B.K. Food Chem.

[B039] Omolo MO, Okinyo D, Ndiege IO, Lwande W, Hassanali A (2004). Repellency of essential oils of some Kenyan plants against *Anopheles gambiae.*. Phytochemistry.

[B040] Pålsson K, Jaenson TGT, Baeckström P, Borg-Karlson AK (2008). Tick repellent substances in the essential oil of *Tanacetum vulgare.*. J Med Entomol.

[B041] Peixoto MG, Costa-Júnior LM, Blank AF, Lima AS, Menezes TSA, Santos DA (2015). Acaricidal activity of essential oils from *Lippia alba* genotypes and its major components carvone, limonene, and citral against *Rhipicephalus microplus.*. Vet Parasitol.

[B042] Rehman JU, Ali A, Khan IA (2014). Plant based products: use and development as repellents against mosquitoes: A review. Fitoterapia.

[B043] Ribeiro FP, Oliveira MS, Feitosa AO, Marinho PSB, Marinho AMR, Andrade EHA (2021). Chemical composition and antibacterial activity of the *Lippia origanoides* Kunth essential oil from the Carajás national forest, Brazil. Evid Based Complement Alternat Med.

[B044] Roditakis E, Roditakis NE, Tsagkarakou A (2005). Insecticide resistance in *Bemisia tabaci* (Homoptera: Aleyrodidae) populations from Crete. Pest Manag Sci.

[B045] Rojas J, Morales A, Pasquale S, Márquez A, Rondón R, Veres K (2006). Comparative study of the chemical composition of the essential oil of *Lippia origanoides* collected in two different seasons in Venezuela. Nat Prod Commun.

[B046] Salvador VF, Morais IML, Leal LLLL, Tamiozo GL, Chagas HDF, Silva IS (2025). Resistance of *Rhipicephalus microplus* to different acaricides in tropical climates: are the laboratory and field results related?. Vet Parasitol.

[B047] Soares BV, Neves LR, Ferreira DO, Oliveira MSB, Chaves FCM, Chagas EC (2017). Antiparasitic activity, histopathology and physiology of *Colossoma macropomum* (tambaqui) exposed to the essential oil of *Lippia sidoides* (Verbenaceae). Vet Parasitol.

[B048] Sousa MAA, Mesquita MLR, Orlanda JFF, Catunda FEA (2020). Chemical composition and phytotoxic activity of *Lippia origanoides* essential oil on weeds. Aust J Crop Sci.

[B049] Souza LM, Fonseca FSA, Silva JCRL, Silva AM, Silva JR, Martins ER (2019). Essential oil composition in natural population of *Lippia origanoides* (Verbenaceae) during dry and rainy seasons. Rev Biol Trop.

[B050] Stashenko EE, Jaramillo BE, Martinez JR (2004). Comparison of different extraction methods for the analysis of volatile secondary metabolites of *Lippia alba* (Mill.) N.E. Brown, grown in Colombia, and evaluation of its in vitro antioxidant activity. J Chromatogr A.

[B051] Stashenko EE, Ruiz C, Muñoz A, Castañeda M, Martínez J (2008). Composition and antioxidant activity of essential oils of *Lippia origanoides* H. B. K. grown in Colombia. Nat Prod Commun.

[B052] Teles S, Pereira JA, Oliveira LM, Malheiro R, Lucchese AM, Silva F (2014). *Lippia origanoides* H.B.K. essential oil production, composition, and antioxidant activity under organic and mineral fertilization: effect of harvest moment. Ind Crops Prod.

[B053] Tozin LRS, Marques MOM, Rodrigues TM (2015). Glandular trichome density and essential oil composition in leaves and inflorescences of Lippia origanoides Kunth (Verbenaceae) in the Brazilian Cerrado. An Acad Bras Cienc.

[B054] Volpato A, Grosskopf RK, Santos RC, Vaucher RA, Raffin RP, Boligon AA (2015). Influence of rosemary, andiroba and copaiba essential oils on different stages of the biological cycle of the tick *Rhipicephalus microplus in vitro.*. J Essent Oil Res.

[B055] Yang JY, Lee HS (2013). Verbenone structural analogues isolated from *Artemesia aucheri* as natural acaricides against *Dermatophagoides* spp. and *Tyrophagus putrescentiae.*. J Agric Food Chem.

[B056] Yang P, Jia M, Zhu L (2021). Acaricidal activity of the essential oil from *Senecio cannabifolius* and its constituents eucalyptol and camphor on engorged females and larvae of *Rhipicephalus microplus* (Acari: ixodidae). Exp Appl Acarol.

[B057] Zhu L, Dai JL, Yang L, Qiu J (2013). In vitro ovicidal and larvicidal activity of the essential oil of *Artemisia lancea* against *Haemonchus contortus* (Strongylida). Vet Parasitol.

